# An olfactory-based Brain-Computer Interface: electroencephalography changes during odor perception and discrimination

**DOI:** 10.3389/fnbeh.2023.1122849

**Published:** 2023-06-15

**Authors:** Marina Morozova, Alsu Bikbavova, Vladimir Bulanov, Mikhail A. Lebedev

**Affiliations:** ^1^Vladimir Zelman Center for Neurobiology and Brain Rehabilitation, Skolkovo Institute of Science and Technology, Moscow, Russia; ^2^VIBRAINT RUS LLC, Moscow, Russia; ^3^Faculty of Mechanics and Mathematics, Moscow State University, Moscow, Russia; ^4^Laboratory of Neurotechnology, I. M. Sechenov Institute of Evolutionary Physiology and Biochemistry, Saint-Petersburg, Russia

**Keywords:** olfaction, Brain-Computer Interface (BCI), neurofeedback, electroencephalography (EEG), respiratory cycle

## Abstract

Brain-Computer Interfaces (BCIs) are devices designed for establishing communication between the central nervous system and a computer. The communication can occur through different sensory modalities, and most commonly visual and auditory modalities are used. Here we propose that BCIs can be expanded by the incorporation of olfaction and discuss the potential applications of such olfactory BCIs. To substantiate this idea, we present results from two olfactory tasks: one that required attentive perception of odors without any overt report, and the second one where participants discriminated consecutively presented odors. In these experiments, EEG recordings were conducted in healthy participants while they performed the tasks guided by computer-generated verbal instructions. We emphasize the importance of relating EEG modulations to the breath cycle to improve the performance of an olfactory-based BCI. Furthermore, theta-activity could be used for olfactory-BCI decoding. In our experiments, we observed modulations of theta activity over the frontal EEG leads approximately 2 s after the inhalation of an odor. Overall, frontal theta rhythms and other types of EEG activity could be incorporated in the olfactory-based BCIs which utilize odors either as inputs or outputs. These BCIs could improve olfactory training required for conditions like anosmia and hyposmia, and mild cognitive impairment.

## Introduction

This perspective article is motivated by our interest in an electroencephalography (EEG)-based BCI that incorporates olfaction. Such a BCI could be used for rehabilitation of patients with impaired olfactory function like patients affected by COVID-19 where one of the clinical symptoms is a sudden deterioration of olfaction, with a greater impact on odor detection threshold than on odor identification (Le Bon et al., 2021). EEG is potentially a useful tool for assessing olfactory processing in humans and adding this information to a BCI control loop. Yet, chemosensory event-related potentials (ERPs) are not as prominent as the commonly used mismatch-negativity and P300 responses in the visual and auditory domains, so it is challenging to analyze them. Analyzing odor-induced changes in EEG rhythms is an alternative to using ERPs. Thus, Schriever et al. (2017) analyzed the induced EEG-power changes during the interval 200–2,000 ms after aroma onset in the frequency band 2–6 Hz, and found that these EEG patterns were informative to distinguish people with olfactory impairments from healthy individuals. Those particular time-frequency parameters were chosen based on the previous studies on the chemosensory responses to trigeminal and olfactory nerve stimulation (Huart et al., 2012).

When designing an olfactory-based BCI, it is important to account for respiration, which is a critical component of olfactory processing in animals (Adrian, 1942; Hobson, 1967; Fontanini et al., 2003; Kepecs et al., 2006; Rojas-Líbano et al., 2014; Frederick et al., 2016; Moberly et al., 2018) and in humans determines the periods when odors are processed and memorized, and affects functional brain connectivity, including effects on non-olfactory areas (Fontanini and Bower, 2006; Arshamian et al., 2018; Corcoran et al., 2018; Perl et al., 2019; Kluger and Gross, 2021; Kluger et al., 2021). Although the respiratory cycle is critical for the analysis of EEG activity in this context, respiratory sensors (Johnson et al., 2006; Merritt et al., 2009; Karacocuk et al., 2019; De Fazio et al., 2021) are often missing in the studies of olfactory processing, with some notable exceptions (Haehner et al., 2011; Noguchi et al., 2011; Wang et al., 2014; Zelano et al., 2016). Overall, notwithstanding the progress made in the investigations of how cortical and subcortical activity is related to the respiratory cycle (Kluger and Gross, 2021; Watanabe et al., 2023), more research is needed for better understanding of how respiration and olfaction are integrated.

Olfactory training (OT), where several odors are actively sniffed by a patient on a daily schedule, is an established approach to olfactory rehabilitation (Hummel et al., 2009; Pekala et al., 2016; Sorokowska et al., 2017; Addison et al., 2021). Adding a BCI approach to OT could be a potentially useful tool to improve rehabilitation (Placidi et al., 2015; Zhang et al., 2021; Ninenko et al., 2022). In such a BCI system, neural patterns occurring during processing of odors are decoded from brain recordings and presented to the user as feedback based on an appropriate sensory modality. Yet, it is critical for implementing this type of BCI that neural responses to odors are well-understood and properly decoded. Currently, it is not well-understood how olfactory processing could be incorporated in the design of a BCIs. In particular, it is not understood which brain signals should be sampled and how they should be analyzed and translated into commands that are transmitted to external devices that assist patients with olfactory disabilities (Alonso-Valerdi et al., 2015). This contrasts with the situation with the most successful BCI implementations that rely on user interaction with visual, auditory or somatosensory stimuli. Accordingly, we propose that incorporating olfaction in BCIs should be explored as a potentially powerful way to modulate brain activity.

Our research program on the olfactory BCIs is based on three considerations. First, we suggest that BCIs that interact with an olfactory environment could be useful for neural technologies targeting emotions and relaxation. Second, we suggest that EEG recordings are suitable for decoding olfaction-representing neural activity, such as modulations of EEG rhythms and P300 responses to odors and/or images that match these odors. Third, we reason that the operation of different types of BCIs could be enhanced by adding odors that enrich the sensory stimuli, for example, adding odors to a virtual environment.

## Theta activity and olfactory processing

To substantiate our idea of an olfactory-based BCI, here we present our results obtained in a study where theta activity changes were detected in participants who perceived and discriminated odors.

The involvement of theta oscillations in olfactory processing has been extensively studied in animals (Bressler and Freeman, 1980; Buonviso et al., 2006; Kepecs et al., 2006; Kay et al., 2009; Rojas-Líbano et al., 2014). In humans, theta oscillations have been linked to odor processing (Jiang et al., 2017) and working memory for odors (Yang et al., 2021). The involvement of beta and gamma oscillations in olfactory processing has been demonstrated in numerous studies in animals (Adrian, 1942; Rosero and Aylwin, 2011; Lockmann et al., 2018) and in humans, as well (Jung et al., 2006; Iravani et al., 2020, 2021; Yang et al., 2022).

**FIGURE 1:**
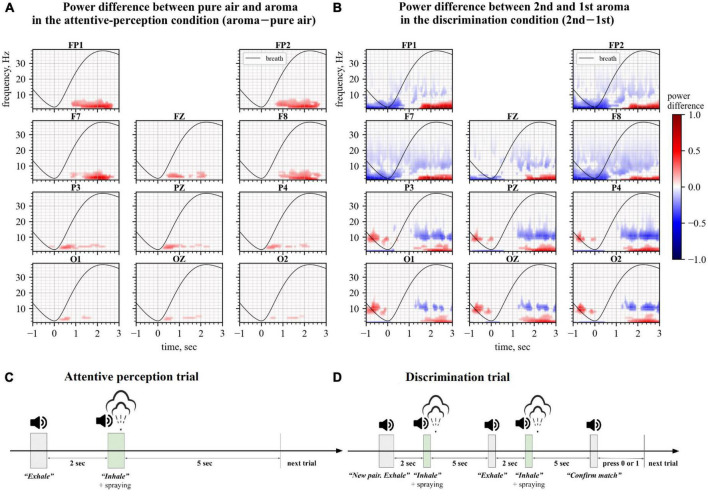
Changes in Electroencephalography (EEG) spectra during the perception (left) and discrimination (right) tasks. Spectral plots represent the power difference where average event-related spectral perturbations (ERSPs) for one condition were subtracted from the ERSPs for another condition. Only statistically significantly different time-frequency regions defined by permutation analysis are shown. The black line represents the average breath cycle. **(A)** Data for the attentive perception task. Here, ERSPs of the no-odor epochs were subtracted from the ERSPs of the odor epochs. **(B)** Data for the discrimination task. ERSPs of the 1st aroma epochs were subtracted from the ERSPs of the 2nd aroma epochs. **(C,D)** Timings of events in trials of attentive perception and discrimination conditions, respectively.

In our experiments, we observed changes in the frontal EEG theta power when healthy participants inhaled different odorants and perceived them (Figure 1A) and when they compared two consecutively presented odors and reported whether or not they were different (Figure 1B).

## An experimental setup for an olfactory-based BCI

In our experimental setup, participants were comfortably seated in front of an Aroma Shooter^§^ diffuser (Aromajoin Corporation, Kyoto, Japan) T mounted 20 cm from the participant’s nose. The diffuser was equipped with six aroma cartridges, namely caramel, grass, orange, pine, smoke, and mint scents (identification numbers S-SW4, S-GN1, N-CT1, N-WD7, S-IM16, and N-HB21, respectively). During the experiment, participants were asked to keep their eyes open. Respiration was measured with a nasal thermometric breath sensor TRSens (MKS, Moscow, Russia).

Electroencephalography data were collected using an NVX-36 amplifier (MKS, Moscow, Russia). Twenty two EEG channels were recorded according to the international “10–20” system with the sampling frequency of 250 Hz using Ag/AgCl electrodes lubricated by an electrode gel. The ground lead was attached to the FCz site. Two reference electrodes were placed on the left and right earlobes. The electrode impedance was kept below 15 kΩ.

Thirteen healthy participants were recruited (six males and seven females; 24.1 ± 5.8 years old, mean ± SD) who performed tasks that required perceiving and discriminating odors. Experiments were approved by the local Ethics Committee of the Skolkovo Institute of Science and Technology, Moscow. The participants gave informed consent to participate in the study. The participants did not have a history of neurological diseases and reported having no significant changes in the sense of smell in the previous 6-months period. All participants were confirmed to be normosmic using the sniffin’ sticks test with 12 items (SST-12).

The participants were native Russian speakers. Accordingly, during the experimental trials, verbal commands were given in Russian using computer audio.

## Perception and discrimination tasks

During the first experimental condition, called attentive perception, participants familiarized themselves with the set of aromas. The condition consisted of 100 trials where their respiration was guided by the commands “Exhale” and “Inhale.” The command “Exhale” was given prior to odor delivery, and the command “Inhale” was issued after an odor was sprayed by the Aroma Shooter^§^ diffuser using a 0.5 s long spray (Figure 1C). The participants were explained that no odor would be delivered in some trials, and half of the trials were odorless. The participants were instructed to pay attention to their olfactory perceptions, but they were not required to name or discriminate the odors being presented. The participants were not given any information regarding the names of the odors.

During the second experimental condition, called discrimination, participants were required to discriminate odors. This condition included 40–60 trials (median 50 trials), each consisting of the following sequence of steps: (1) an auditory command “New pair”; (2) the commands “Exhale” and “Inhale” followed by a 0.5 s long spray of the first odorant from the diffuser; (3) a 5 s period for the odor to dissipate; (4) the commands “Exhale” and “Inhale” followed by a 0.5 s spray of the second odorant; (5) the command “Confirm match” after which the participant pressed “1” or “0” on the keyboard to report that the first and the second odorants were identical or different, respectively (Figure 1D). The number of trials with the identical odorants in the pair was equal to the number of trials with different odorants.

## Analyses of EEG responses

**FIGURE 2:**
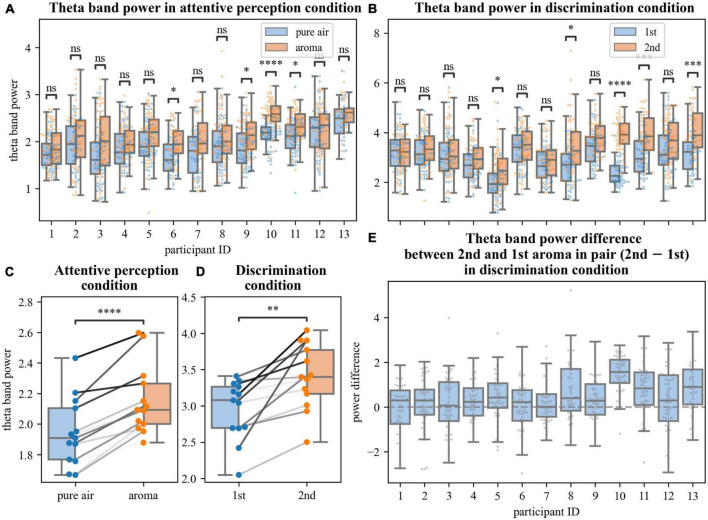
Analyses of changes in theta power. **(A,B,E)** Average power in statistically significantly different time-frequency regions defined by permutation analysis. Each point represents the average power in an epoch **(A,B)** or difference in average theta powers between epochs corresponding to the 2nd and 1st aromas in pair in the discrimination task **(E)**. Different pairs of boxplots divided by conditions [pure air and aroma, **(A)**; 1st and 2nd aromas in pair, **(B)**] and single boxplot **(E)** correspond to different participants. **(C,D)** Averaged for each participant theta power in perception **(C)** and discrimination **(D)** tasks in different conditions. Data points of the same participants are connected by gray lines. ns: 5.00e–02 < *p* ≤ 1.00e + 00; *1.00e–02 < *p* ≤ 5.00e–02; ^**^1.00e–03 < *p* ≤ 1.00e–02; ^***^1.00e–04 < *p* ≤ 1.00e–03; ^****^p ≤ 1.00e–04.

EEG data were split into 4 s long epochs for processing. Each epoch started 1 s before the beginning of the inhale and ended 3 s after it. The beginning of the inhale was determined based on the measurements from the breath sensor. Each epoch was z-score standardized prior to the time-frequency analysis. Time-frequency analysis of the epochs was performed using the Morlet continuous wavelet transform (CWT) with the initial spread of the Gaussian wavelet set at 2.5/πω_0_ (where ω_0_ is the central frequency of the wavelet). CWT was applied to a single epoch in the 1–40 Hz frequency band. To calculate event-related spectral perturbations (ERSPs), average time-frequency maps were calculated for the absolute values of the signal. Cluster-level statistical permutation test (Maris and Oostenveld, 2007) with Kruskal-Wallis H-statistic as the test statistic was applied to all collected epochs to compare time-frequency maps for odor and no-odor trials of the Random condition and the first and second odorants of the Matrix condition. For the detected statistically significant clusters, average power across all electrodes was calculated for each epoch. Verification of distribution normality was carried out using D’Agostino-Pearson test, non-parametric tests were used in case of deviations from normality. We compared the sets of average cluster powers for each participant using Mann-Whitney-Wilcoxon test with Bonferroni correction (Figures 2A, B), and also compared averaged across trials cluster powers for each participant using *t*-test for paired samples (Figures 2C, D).

A cluster-level statistical permutation test was applied to compare CWT time-frequency maps of the odor and no-odor trials for the attentive-perception exercise. We found a significant increase in the frontal theta-range activity during the odor presentation trials as compared to the no-odor trials (cluster-level statistical permutation test, *p*-value = 0.03). This increase occurred during the interval from 1.5 to 2.5 s relative to the inhale onset (Figure 1A). We calculated and averaged across trials mean power in the statistically significant theta-range cluster and found a statistically significant difference in the mean theta power when the odor and no-odor trials were compared. A 10.5% increase in the theta power was observed in odor trials on average (no deviations from normality; *t*-test paired samples, *p*-value = 5.615e–06, t-statistic = –7.691; Figure 2C).

We compared CWT time-frequency maps for the first and second odors epochs of the discrimination condition. We found a significant increase in the frontal theta activity and besides the changes in the theta activity, we found a slight decrease in beta power during the second odor (cluster-level statistical permutation test, *p*-value = 0.1, Figure 1B). The calculated and averaged across trials mean power in the theta-range cluster was statistically significantly higher during the second odor (no deviations from normality; *t*-test paired samples, *p*-value = 2.340e–03; t-statistic = –3.843; Figure 2D) with a 14.2% increase on average.

## Discussion

Here we proposed developing an olfactory-based BCI. As the first step toward such a system, we constructed an experimental setup where subjects perceived and discriminated consecutively presented odors while their EEG activity was sampled. Under these experimental conditions, we found a significant increase in the frontal theta power during the period when healthy participants inhaled odorants as compared to inhaling air without any odorants for all participants (Figures 1A, 2A, C). We also found a significant increase in the theta power when the participants compared two consecutively presented odorants and reported whether or not they were different (Figures 1B, 2B, D, E). These EEG changes are suitable for decoding characteristics of olfactory processing using a BCI.

The correlations of the theta power with, firstly, the presence/absence of aroma and, secondly, cognitive load provide the opportunity to use the theta power as an objective EEG-based metric for a machine learning model used in a BCI. The model can be adjusted to give an objective score to estimate the threshold of perception of a smell and the level of focus to an olfactory task. We suppose that changes in theta power during the odor perception throughout the long-term olfactory rehabilitation process may also be a monitored metric to control rate and degree of olfactory system recovery.

These results could be extended to building an olfactory-based BCI where frontal theta-power changes related to perceived odors are converted into an output signal (e.g., visual feedback). Thus, there may be a BCI-based training of olfactory system where the participant’s task will be to focus on perceived aromas and produce the most significant visual feedback (for example, circle with size or/and transparency defined by the power of theta response during the odor perception). Conversely, frontal theta-power could be converted into an olfactory neurofeedback where odor type and intensity represent changes in the EEG so that, for example, the bigger the theta power during the odor perception the smaller amount of aroma will be sprayed in the next iteration. Such BCIs could be useful for rehabilitation of people with olfactory disabilities. Additionally, rehabilitation of age-related mild cognitive impairment is also a potential field of application.

## Data availability statement

The raw data supporting the conclusions of this article will be made available by the authors, without undue reservation.

## Ethics statement

The studies involving human participants were reviewed and approved by Local Ethics Committee of the Skolkovo Institute of Science and Technology. The patients/participants provided their written informed consent to participate in this study.

## Author contributions

VB contributed to constructing the experimental setup and writing the software. MM, AB, and ML equally contributed to the development of the experiment design, data collection and analysis, and writing of the manuscript. All authors contributed to the article and approved the submitted version.
